# The “fossilized” mitochondrial genome of *Liriodendron tulipifera*: ancestral gene content and order, ancestral editing sites, and extraordinarily low mutation rate

**DOI:** 10.1186/1741-7007-11-29

**Published:** 2013-04-15

**Authors:** Aaron O Richardson, Danny W Rice, Gregory J Young, Andrew J Alverson, Jeffrey D Palmer

**Affiliations:** 1 Department of Biology, Indiana University, Bloomington, IN, 47405, USA; 2 DuPont Pioneer, Wilmington, DE, 19880, USA; 3 Department of Biological Sciences, University of Arkansas, Fayetteville, AR, 72701, USA

**Keywords:** Angiosperm, RNA editing, Molecular evolution, Substitution rate, Intracellular gene transfer, Mitochondrial genome

## Abstract

**Background:**

The mitochondrial genomes of flowering plants vary greatly in size, gene content, gene order, mutation rate and level of RNA editing. However, the narrow phylogenetic breadth of available genomic data has limited our ability to reconstruct these traits in the ancestral flowering plant and, therefore, to infer subsequent patterns of evolution across angiosperms.

**Results:**

We sequenced the mitochondrial genome of *Liriodendron tulipifera*, the first from outside the monocots or eudicots. This 553,721 bp mitochondrial genome has evolved remarkably slowly in virtually all respects, with an extraordinarily low genome-wide silent substitution rate, retention of genes frequently lost in other angiosperm lineages, and conservation of ancestral gene clusters. The mitochondrial protein genes in *Liriodendron* are the most heavily edited of any angiosperm characterized to date. Most of these sites are also edited in various other lineages, which allowed us to polarize losses of editing sites in other parts of the angiosperm phylogeny. Finally, we added comprehensive gene sequence data for two other magnoliids, *Magnolia stellata* and the more distantly related *Calycanthus floridus,* to measure rates of sequence evolution in *Liriodendron* with greater accuracy. The *Magnolia* genome has evolved at an even lower rate, revealing a roughly 5,000-fold range of synonymous-site divergence among angiosperms whose mitochondrial gene space has been comprehensively sequenced.

**Conclusions:**

Using *Liriodendron* as a guide, we estimate that the ancestral flowering plant mitochondrial genome contained 41 protein genes, 14 tRNA genes of mitochondrial origin, as many as 7 tRNA genes of chloroplast origin, >700 sites of RNA editing, and some 14 colinear gene clusters. Many of these gene clusters, genes and RNA editing sites have been variously lost in different lineages over the course of the ensuing ∽200 million years of angiosperm evolution.

## Background

Angiosperm mitochondrial genomes are remarkably variable in both structure and sequence content. They range in size by nearly two orders of magnitude, from approximately 220 kb [1] to >11,000 kb [2]; most of this size variation is due to differing amounts of noncoding DNA. Gene and intron content also vary considerably among species, ranging from 32 to 67 genes and 18 to 25 introns [3], some of which are encoded in discontinuous fragments that require *trans*-splicing [4]. The variable gene content reflects a pattern of differential losses and functional transfers to the nucleus across angiosperms [5]. In addition, no sequenced angiosperm mitochondrial genome contains a full set of native tRNAs necessary for the translation of its full gene complement [6]. Moreover, tRNA content varies widely among species, with each genome containing some mixture of tRNAs of mitochondrial, plastid and possibly bacterial origin [3,7,8]. Missing tRNAs are encoded in the nucleus and imported from the cytosol. In general, genes that do remain in angiosperm mitochondrial genomes evolve slowly in sequence relative to plant nuclear and chloroplast genomes [9]. Substitution rates are, however, hundreds of times higher in some lineages [10-12]. Gene order also varies substantially among species, at the level of a plant family [13,14], and in some cases, even between cultivars of the same species [15]. Although the arrangement of gene clusters varies [16-18], several syntenic blocks of genes are conserved all the way back to their bacterial progenitors [19,20].

RNA editing is another source of variability among angiosperm mitochondrial genomes. Most mitochondrial protein genes are subject to some level of C-to-U editing in the messenger RNA [21]. Nearly all edits are nonsynonymous (for example, 87% of edits in *Arabidopsis*), resulting in an amino acid translation different from that encoded by the genome sequence; in many cases, this change restores the evolutionarily conserved amino acid residue [22-24]. The level of editing across angiosperms varies by more than a factor of two. Within eudicots, the number of empirically determined editing sites across 32 protein-coding genes ranges from 430 sites in *Arabidopsis* (Brassicales) to just 189 sites in *Silene noctiflora* (Caryophyllales) [25]. Comparative analyses point to a pattern of progressive loss, not gain, of editing sites across angiosperms [25-27]. This pattern of loss appears to predate the origin of angiosperms, as there are increasing levels of RNA editing observed in earlier branching lineages, from the gymnosperm *Cycas* (approximately 1,100 sites) [28,29], which shared a common ancestor with angiosperms more than 300 million years ago (mya) [30,31], to the lycophyte *Isoetes* (approximately 1,500 sites) [32], which last shared a common ancestor with angiosperms approximately 400 mya [30,31].

Although complete sequencing of plant mitochondrial genomes has greatly informed our understanding of their evolution in angiosperms, these efforts have covered a relatively narrow phylogenetic breadth of species [3]. At the time of this study, 39 angiosperm mitochondrial genomes were available in GenBank, comprising 35 species, 25 genera, 16 families and 13 taxonomic orders. Current estimates place the total diversity of angiosperm at some 250,000 species distributed across 13,000 genera, 444 families and 68 orders [33]. The angiosperm phylogeny is divided roughly into eight major groups [34] (Additional file 1: Figure S1), and all currently sequenced angiosperm mitochondrial genomes come from just two of these groups, the monocots and eudicots. Sampling within these lineages is highly biased towards crop plants, for example, 4 of the 12 monocot genomes come from different maize cultivars and 10 are from a single family, the grasses (Poaceae). The magnoliids, the third largest angiosperm lineage with approximately 10,000 species, has been unsampled until now. This gap, along with the important “early-diverging” phylogenetic position of magnoliids within angiosperms, motivated our decision to sequence the mitochondrial genome of the tulip tree, *Liriodendron tulipifera*. Beyond representing a diverse and understudied lineage, the *Liriodendron* mitochondrial genome is the first from an angiosperm outside of the monocot and eudicot lineages, making it possible to polarize differences between these two groups and thereby offer new insights into the ancestral properties of angiosperm mitochondrial genomes. In addition, previous work identified *Liriodendron* as having among the lowest rates of silent substitution within flowering plants [10], as well as a large and presumably “full” complement of genes [5]. Together, these data suggest that the overall state of the ancestral angiosperm mitochondrial genome may be less obscured by subsequent change in *Liriodendron* compared to other lineages with higher rates of mutation and gene loss.

The goal of this study was to use the *Liriodendron* mitochondrial genome to better understand the evolution of gene content, substitution rate, RNA editing and gene order across flowering plants. To more precisely characterize the substitution rate in the genome, we added comprehensive gene sequence data for two other magnoliids, *Magnolia stellata* and the more distantly related *Calycanthus floridus*. These data, along with the nearly full set of empirically determined RNA-edited sites in *Liriodendron* protein coding genes, showed that the *Liriodendron* mitochondrial genome has evolved exceptionally slowly, with the lowest known genome-wide absolute silent substitution rate, a full set of protein-coding genes, high levels of RNA editing, and retention of ancestral gene clusters.

## Results

### Genome size and content

**Figure 1:**
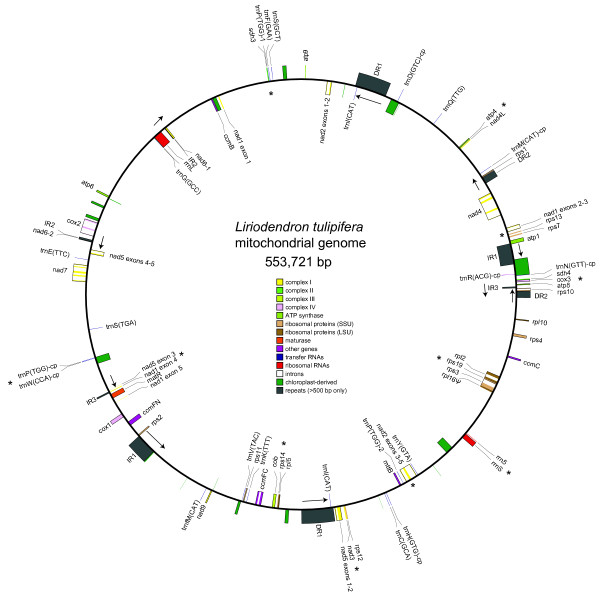
**The mitochondrial genome of *Liriodendron tulipifera.*** Displayed as a circle, but recognizing that the structure is likely to be much more complex *in vivo*[35]. Direct (DR) and inverted (IR) repeats longer than 500 bp and with >99% sequence identity are numbered, with arrows denoting their relative orientation. Genes from the same protein complex are similarly colored, introns are white, and plastid-derived sequences are green and unlabeled. Genes shown on the inside and outside of the circle are transcribed clockwise and counter-clockwise, respectively. Potentially functional tRNAs of plastid origin are noted with a “–cp” suffix. Asterisks identify colinear gene clusters inferred to be present in the ancestral angiosperm mitochondrial genome (see Figure 6 and text for more detail). The map was modified from the output of OGDRAW [37].

The *Liriodendron tulipifera* mitochondrial genome assembled into a single, circular-mapping molecule of length 553,721 bp, with five pairs of direct and inverted repeats >500 bp in length, all of the 41 protein-coding and three rRNA genes inferred to have been present in the ancestral flowering plant mitochondrial genome [3], 13 intact tRNAs of mitochondrial origin, and seven intact and potentially functional chloroplast-derived tRNAs (Figure 1, Additional file 1: Table S1). Recognizing the tenuous link between circular genome assemblies and the structure of plant mitochondrial genomes [35,36], the genome likely exists in different conformations *in vivo*. The *Liriodendron* mitochondrial genome is moderate in size compared to other angiosperms; the median mitochondrial genome size for 28 angiosperms in our analysis (Additional file 1: Figure S2) is 484 kb (500 kb and 431 kb for monocots and eudicots, respectively). Due to its exceptional retention of genes, the total number of nucleotides allocated to intact protein-coding genes in *Liriodendron* (34,449 bp) exceeds that in all other sequenced flowering plant mitochondrial genomes (Additional file 1: Figure S2). Still, protein-coding genes, *cis*-spliced introns, rRNA and tRNA genes together comprise just 14% of the genome (Additional file 1: Table S1).

The 20 *cis*-spliced introns in *Liriodendron* are among the longest in seed plants [38], comprising >35 kb of total sequence (Additional file 1: Figure S2). Among the sequenced angiosperm mitochondrial genomes, only *Liriodendron* and *Vitis* contain the full set of 20 *cis*-spliced introns that are variously present in other angiosperms. With just 13 *cis-*spliced introns, *Silene latifolia* is the most depauperate in this regard [39]. *Liriodendron* also contains the same five *trans*-spliced introns found in all sequenced angiosperm mitochondrial genomes. *Liriodendron* lacks the derived intron states found in some angiosperm mitochondrial genomes. That is, it contains both of the frequently lost *cox2* introns [40], it lacks the horizontally invasive *cox1* intron [41,42], and it has not experienced either of the two *trans*-splicing fractures in the fourth intron of *nad1* that have occurred multiple times over the course of angiosperm evolution [43]. Finally, the *rps3* gene of *Cycas* and other gymnosperms contains a second intron [44,45] that is not present in *Liriodendron*, suggesting that if the intron was gained early in seed plant evolution and lost in angiosperms [45], the loss occurred prior to the divergence of the magnoliids from the common ancestor of monocots and eudicots.

**Figure 2:**
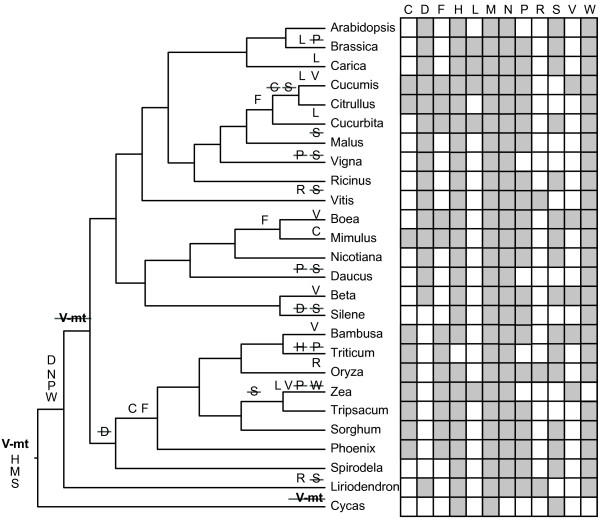
**Proposed evolutionary history of plastid-derived tRNA genes found in angiosperm mitochondrial genomes.** Also shown, in bold, is one native mitochondrial tRNA gene, *trnV(TAC)-mt*. Open text on a branch denotes a gain of that gene, whereas strikethrough indicates the loss of that gene. Gray rectangles in the grid on the right hand side indicate presence of a given full length tRNA in each mitochondrial genome. Of the sequenced *Silene* mitochondrial genomes, *S. latifolia* was used here. Key: C = *trnC(GCA)-cp*, D = *trnD(GTC)-cp*, F = *trnF(GAA)-cp*, H = *trnH(GTG)-cp*, L = *trnL(CAA)-cp*, M = *trnM(CAT)-cp*, N = *trnN(GTT)-cp*, P = *trnP(TGG)-cp*, R = *trnR(TCT)-cp*, S = *trnS(GGA)-cp*, **V-mt** = *trnV(TAC)-mt*, W = *trnW(CCA)-cp*.

The pattern of long-term gene retention in *Liriodendron* also extends to the tRNA genes. Plant mitochondrial genomes contain tRNAs of diverse origin – native mitochondrial, plastid [7], and one, *trnC(GCA)*, that appears to have been horizontally transferred from bacteria [8]. The *Liriodendron* mitochondrial genome possesses intact copies of 12 of the 13 tRNA genes of mitochondrial origin found variously across angiosperms, as well as a truncated remnant of the other, *trnD*(*GTC).* Interestingly, the *Liriodendron* mitochondrial genome contains an additional mitochondrial tRNA, *trnV(TAC)*, which is so far unknown from monocot and eudicot mitochondrial genomes and is present only as a truncated pseudogene in the gymnosperm *Cycas*[28]. Thus, although previous evidence pointed to the pseudogenization and eventual loss of *trnV(TAC)* early on in the evolution of seed plants, it now appears likely there were independent losses in both *Cycas* and the common ancestor of monocots and eudicots (Figure 2). *Liriodendron,* therefore, contains more intact, native mitochondrial tRNAs – 13 of them – than any other sequenced flowering plant mitochondrial genome. Many eudicots (*Carica*, *Citrullus*, *Vitis* and *Nicotiana*) are missing only an intact *trnD(GTC)* and *trnV(TAC)*[39]. *Liriodendron* does not contain the bacterial-like *trnC(GCA)* found in *Beta*[46], *Vigna*[40], *Citrullus*[14] and *Spinacea*[8], supporting a later, likely horizontal origin of this tRNA early on in eudicot evolution [8]. As in *Arabidopsis* and cucurbits, there is a modified plastid-like *trnI* immediately downstream of the *ccmC* gene in *Liriodendron*, a “t-element” that facilitates transcriptional termination [14,47]. This feature, too, likely was present in the ancestral angiosperm mitochondrial genome.

### Plastid-derived tRNAs

Non-functional plastid-derived sequences comprise 0.5 to 11.5% of the genome in previously sequenced angiosperm mitochondrial genomes [14,40], and transferred plastid sequences comprise about 5% in *Liriodendron*. Although most transferred plastid sequences are presumed to be “dead on arrival”, there are several widely conserved and putatively functional plastid-derived tRNAs in angiosperm mitochondrial genomes [7]. In any given genome, however, it is difficult to determine the precise number of functional tRNAs of plastid origin due to the ongoing, high rate of sequence transfer from the plastid. The most straightforward cases of likely functional plastid-derived tRNAs are those surrounded by native mitochondrial sequence, which suggests that purifying selection has maintained them even as their co-transferred flanking sequences were ameliorated via genome rearrangement and sequence degradation. In contrast, other tRNAs are embedded within larger tracts of recently transferred and presumably nonfunctional plastid sequence, suggesting that their presence is only coincidental [14]. Four of the six intact plastid-derived tRNAs in *Liriodendron* are located within larger tracts of plastid DNA. The two solitary plastid-derived tRNAs, *trnH(GTG)-cp* and *trnM(CAT)-cp*, are also present in *Cycas* and most other angiosperms (Figure 2), supporting the hypothesis that they are functional and ancestrally present. Based on their presence in a diverse set of other angiosperms, it appears likely that *trnN(GTT)-cp, trnP(TTG)-cp, trnW(CCA)-cp* and *trnD(GTC)-cp* are functional as well (Figure 2). The sporadic presence of *trnC(GCA)-cp*, *trnF(GAA)-cp*, *trnL(CAA)-cp* and *trnV(GAC)-cp* across angiosperms, along with their absence from *Liriodendron* and *Cycas,* likewise suggests that these tRNAs represent more recent acquisitions and were, therefore, not present in the ancestral angiosperm mitochondrial genome.

**Figure 3:**
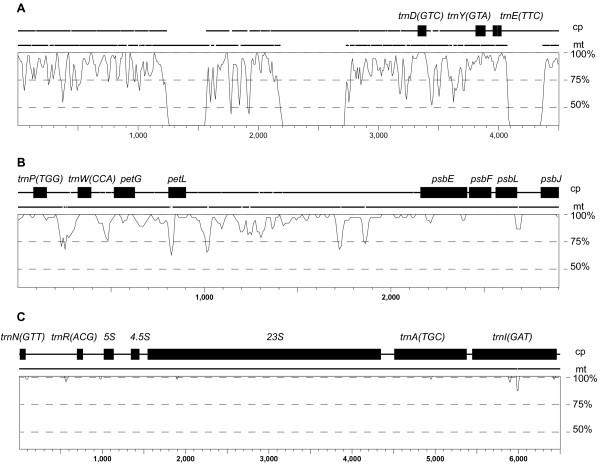
**Percent identity plots for three different stretches of plastid DNA present in the *Liriodendron *mitochondrial genome. A**) 3,546 bp fragment with 84.4% average pairwise identity, excluding gaps; **B**) 2,859 bp fragment, 95.2% average pairwise identity, excluding gaps; **C**) 6,661 bp fragment, 99.9% average pairwise identity, excluding gaps. Above each graph is a linear representation of the *Liriodendron* chloroplast (cp) and cognate mitochondrial (mt) sequence, with line breaks indicating indels in one sequence relative to the other, and labeled genes represented by black rectangles. The vertical axis shows the percent identity scale and the horizontal axis shows the scale for the pairwise alignment; note the different horizontal scale for each graph.

Based on our analysis, seven plastid-derived tRNAs were likely present in the ancestral angiosperm mitochondrial genome, four of which appear to have been acquired after the split between angiosperms and gymnosperms (Figure 2). In light of the slow rate of gene loss and gene cluster rearrangement (see “Gene cluster conservation” below) in *Liriodendron*, we examined the flanking sequences around these four plastid-derived tRNAs for any remnants from the original transfer events that would have occurred roughly 150 to 300 mya, after the gymnosperm–angiosperm split, but prior to the radiation of angiosperms (Figure 3). The four tRNAs are located within three larger plastid-derived regions that vary in their level of divergence with their progenitor plastid sequence, probably, at least partly, due to differences in the timing of their respective transfers. The oldest, based on sequence divergence, is a 3.5 kb stretch of plastid-like sequence containing the putatively functional *trnD(GTC)-cp* that is present in most eudicot mitochondrial genomes but absent from monocots and *Cycas* (Figure 3A). Within the tRNA itself, there is 100% sequence identity between the plastid and mitochondrial copies in *Liriodendron*, compared to just 84% average sequence identity in the full 3.5 kb fragment. The *trnY* and *trnE* genes in these flanking regions are clearly nonfunctional due to multiple indels in each of them. These two tRNAs are present in scattered eudicots (*Vitis*, *Nicotiana* and cucurbits), though they are not linked with *trnD(GTC)-cp*. The *trnP(TTG)-cp* and *trnW(CCA)-cp* genes present colinearly in most angiosperm mitochondrial genomes, but absent from *Cycas*, are located in a second 2.9 kb plastid-like fragment in *Liriodendron* (Figure 3B). Again, although the mitochondrial and plastid copies of each gene are identical, similarity in the flanking regions drops off to 95% average pairwise identity. A third, 6.6 kb fragment (Figure 3C) that harbors *trnN(GTT)-cp,* which is present in all sequenced angiosperm mitochondrial genomes but absent from *Cycas* (Figure 2), has nearly 100% pairwise sequence identity between the plastid and mitochondrial copies in *Liriodendron*, suggesting that this region was more recently transferred.

### Substitution rates

**Figure 4:**
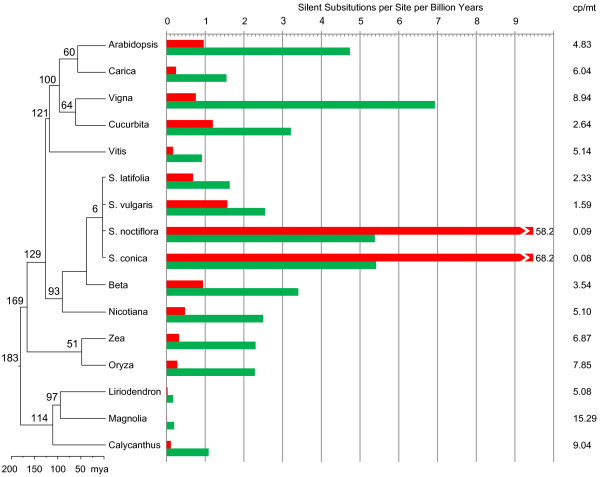
**Variation in the absolute rates of silent substitution in plastid (green) and mitochondrial (red) genomes across diverse angiosperms.** The tree was rooted on *Cycas*, which was subsequently removed for presentation clarity. Branch-specific absolute silent substitution rates (per billion years) for the mitochondrial and chloroplast genomes are in red and green, respectively. Rate estimates for *Silene* were taken from previously published reports [2,48]. Confidence intervals of 95% derived from the error in estimating branch-specific synonymous substitution rates are given in Additional file 1: Table S3.

We used the complete genome sequence from *Liriodendron* and gene sequences from two other magnoliids, *Magnolia stellata* (Magnoliaceae) and *Calycanthus floridus* (Lauraceae), to examine genome-wide rates of silent substitution across angiosperms (>16 kb of concatenated gene sequence from 13 taxa). When scaled to a fossil-calibrated chronogram, *Liriodendron* had an absolute rate of 0.035 silent substitutions per billion years (ssb), while at just 0.013 ssb, its sister genus, *Magnolia*, had the slowest rate of all sequenced angiosperms (Figure 4). *Silene conica* had the highest rate (68.2 ssb), which is roughly 5,000 times faster than *Magnolia*.

Within the genus *Magnolia*, the mitochondrial silent substitution rate is unmeasurable from these data, with no silent substitutions observed across six genes (*atp1*, *nad3*, *nad5*, *rps4, rps12, rps13*; 5,718 bp) for which multiple high-quality sequences are available (from GenBank and this study) for at least two species within the genus: *M. figo*, *M. grandiflora*, *M. tripetala*, *Magnolia x soulangeana* and *M. stellata*. These taxa last shared a common ancestor an estimated 48 to 54 mya [49], and the mitochondrial genes appear to have remained frozen in time since then. Similarly, in the four genes (*atp1*, *matR*, *nad5* and *rps3*; 7,098 bp) for which sequence is available for both *L. tulipifera* and its sister species, *L. chinense*, there has been but a single silent substitution between them in the 10 to 16 million years since their split [50], pointing again to an apparently very low silent substitution rate in this lineage.

We also examined substitution rates in angiosperm plastid genes, which evolve at a rate intermediate between genes in the mitochondrial and nuclear genomes [9,13,51,52]. Although plastid silent substitution rates also vary across species, the patterns are generally more complex, in that the rate increases might only be due to a subset of the genes and there are increases in nonsynonymous rates in some cases as well [48,53]. Among the 16 species in this study, *Liriodendron* and *Magnolia* have the slowest evolving plastid genes as well, at 0.18 and 0.20 ssb, respectively, which are slower than most angiosperm mitochondrial genes (Figure 4). At 9.0 ssb, *Vigna* and *Calycanthus* had the highest rates of chloroplast sequence evolution, some 50 times faster than in the *Liriodendron* chloroplast. The mitochondrial and chloroplast silent substitution rates are roughly correlated within species. If we omit the two “superfast” mitochondrial lineages in *Silene*, a linear regression of chloroplast-to-mitochondrial silent substitution rates has a slope of 2.22 (R^2^ = 0.35). The mean ratio of this sample is 5.28 (+/− 3.87) chloroplast:mitochondrial substitutions. Previous studies have estimated mitochondrial silent substitution rates are between two and four times lower than plastid rates in angiosperms [9,13,51,52]. However, in light of new data from taxa with more extreme rates, these studies appear to have underestimated both the central tendency and the variability, as the ratio of mitochondrial to plastid substitution rate ranges from 0.08 to 15.29 in our data (Figure 4).

### RNA editing

To examine the evolution of RNA editing across angiosperms, we sequenced 30,327 nucleotides of cDNA from 38 protein genes in *Liriodendron* (comprising 88% of the protein-coding sequence in the genome) and aligned DNA and cDNA sequences for 11 angiosperms to identify editing sites. *Liriodendron* is the most highly edited of any angiosperm mitochondrial genome with comprehensive empirical RNA editing data. Among the 11 angiosperms used in this analysis, which included one monocot, nine eudicots, and *Liriodendron*, we found a total of 1,086 sites across 34 genes that were edited in at least one taxon (Additional file 1: Table S2). *Liriodendron* was edited at 781 of the 1,086 sites, nearly 70% more than the next most heavily edited taxon, *Nicotiana*, which has 463 edit sites. *Liriodendron* is exceptional for its large number of unique edit sites, 138, relative to the 10 other species in this analysis; the next highest is *Oenothera* with 61 while *Silene* has only 2 unique sites (Additional file 1: Table S2).

**Figure 5:**
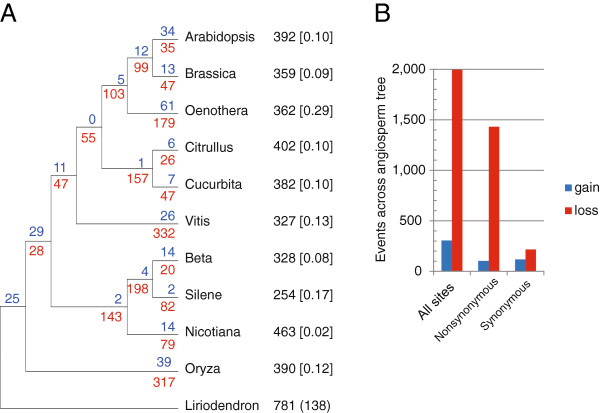
**The evolution of RNA editing level across angiosperms.** (**A**) Inferred gains (blue) and losses (red) of RNA editing sites using Dollo parsimony. The total number of edited sites determined across an alignment of 30,327 bp and 38 protein genes is listed next to each taxon. The proportion of missing data due to gene loss or lack of cDNA sequence is shown in brackets. The number of edit sites unique to *Liriodendron*, based on this current sample of angiosperms with empirically determined RNA editing data, is shown in parentheses. Parsimony cannot distinguish whether these sites were gained in the *Liriodendron* lineage, or present ancestrally and lost on the branch leading to the rest of the angiosperms (thus, no losses are noted on that branch as well). (**B**) Edit sites partitioned by whether they occur at a nonsynonymous or synonymous position in the codon. Edit sites in codons with multiple edits or where *Liriodendron* encoded a G or A were omitted in the partitioned data; therefore, the partitioned sites sum to less than the total number of sites in the study. ‘Silene’ is *S. latifolia*.

Because *Liriodendron* is sister to the lineage containing all other species in the analysis (Figure 5), we could not distinguish between gains on the branch leading to *Liriodendron* from ancestral edit sites retained in *Liriodendron* and lost in the common ancestor of monocots and eudicots. We attempted to use empirically-determined RNA edit sites in *Cycas*[29] to polarize changes in *Liriodendron*, but *Cycas* is missing data for 575 of the 1,086 sites in our alignment. Moreover, more than half of the edit sites in *Cycas* that are in our alignment were not edited in angiosperms. Therefore, because we could not reliably polarize gains and losses at the root of the tree, we infer that the ancestral angiosperm shared on the order of 643 to 781 of the edit sites in the genes analyzed here. Although gains of edit sites have occurred, loss predominates across angiosperms in our study (Figure 5A), which is consistent with the previous pervasive-loss models [25-27]. When edits at all sites (synonymous and nonsynonymous) were considered, we inferred 1,994 losses and 305 gains across the tree, a ratio of nearly 7:1 (Figure 5B). Consistent with previous observations [21], 82% of edit sites were predicted to change the translated amino acid. Although editing of synonymous sites was less common overall, gains were more frequent at synonymous sites. The loss-to-gain ratios at synonymous and nonsynonymous sites were approximately 2:1 and 14:1, respectively.

### Gene cluster conservation

**Figure 6:**
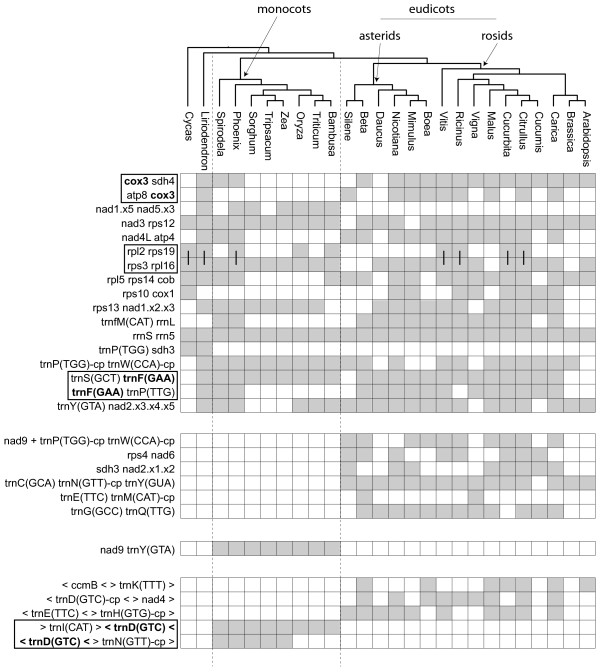
**Colinear mitochondrial gene clusters across angiosperms.** The cladogram above shows the phylogenetic relationships among these sequenced mitochondrial genomes [33]. Gray rectangles denote the presence of a gene cluster, even if one of the genes is a pseudogene. Clusters composed of more than two genes are split into two-gene subclusters and boxed together, with the common gene of both in bold. In the case of the four gene cluster *rpl2-rps19-rps3-rpl16*, there are two two-gene subclusters, which themselves can be colinear. In these cases, a vertical bar connects the two gray rectangles in the right panel. In the top panel are clusters inferred to have been present in the common ancestor of angiosperms. The bottom three panels show clusters inferred to have been present in the common ancestor of eudicots or monocots, but not in the ancestral angiosperm. The bottom panel contains clusters where the genes involved are encoded on opposite strands in the genome, whereas all clusters in the top three involve genes on the same strand and orientation. Genes in the bottom panel are noted as being in the forward strand (>) or reverse strand (<).

We examined the *Liriodendron* mitochondrial genome for the presence of conserved gene clusters, which we define as two or more adjacent genes in *Liriodendron* that are shared, in the same orientation, with at least one other species in our analysis. In all, we found 12 such gene clusters in *Liriodendron* (Figure 6). Two additional clusters that were probably present early in angiosperm evolution are missing in *Liriodendron*: *rps10–cox1* (present in *Cycas* and many eudicots) and *trnfM–rrnL* (present in mosses, liverworts, lycophytes, eudicots and some monocots). *Cycas* is missing eight of the 14 clusters we inferred to have been present in the ancestral angiosperm mitochondrial genome. One conserved cluster, which joins the fifth and final exon of *nad1* with the third exon of the *trans*-spliced *nad5* gene, is shared between monocots and *Liriodendron* to the exclusion of eudicots. Another cluster (*atp8–cox3*–*sdh4*) is shared between *Liriodendron* and eudicots to the exclusion of monocots (Figure 6). Without *Liriodendron* as an outgroup, it was impossible to polarize whether these clusters represented ancestral or derived gene arrangements. While *Liriodendron* shows the greatest overall level of conservation of the inferred ancestral angiosperm gene clusters – retaining 12 out of 14 of them – several other species have retained equally as many (12/14; *Citrullus*) or nearly as many (11/14; *Phoenix*, *Vitis*, *Carica* and *Ricinus*), though the combinations of conserved clusters vary among species. At the other extreme, the grasses, *Silene*, *Arabidopsis* and *Brassica* have retained only five or six ancestral gene clusters. The *Liriodendron* data also allowed us to assign nine conserved clusters as restricted to eudicots at various phylogenetic depths, as well as two restricted to monocots, assuming no loss in *Liriodendron* (Figure 6). Although we did not constrain our analysis to consider only clusters encoded on the same strand, this was the case for all but three conserved colinear clusters in eudicots, and one within monocots (Figure 6, bottom panel).

## Discussion

We sequenced the mitochondrial genome of *Liriodendron tulipifera*, the first from the large (>10,000 species) magnoliid lineage, to fill an important phylogenetic gap and provide an outgroup for comparison to the previously studied monocot and eudicot lineages. The phylogenetic position of *Liriodendron* allowed us to polarize changes in monocots and eudicots, leading to a more detailed understanding of the patterns of loss of RNA editing, gains of plastid tRNAs, and gene cluster conservation across flowering plants. These efforts were bolstered by the fact that the *Liriodendron* mitochondrial genome evolves exceptionally slowly in terms of gene sequence, content and order, allowing an unprecedented look into the early evolution of plant mitochondrial genomes. Thus, in many striking ways, *Liriodendron* has a “fossilized” mitochondrial genome, having undergone remarkably little change over the last ∽100 million years.

### Insights into the acquisition of plastid-derived tRNAs

The evidence presented here points to a different evolutionary history of mitochondrial plastid-derived tRNAs in angiosperms than previously postulated [16,54], generally pushing back their origins earlier in flowering plant evolution (Figure 2). Whereas Wang *et al.*[54] posited a recent origin of *trnP(TGG)-cp* on the branch leading to *Nicotiana*, its presence in monocots, eudicots and now magnoliids (Figure 2) suggests that its acquisition likely predated the common ancestor of these three lineages. Similarly, the presence of *trnD(GTC)-cp* in *Liriodendron* likely pushes the origin of that tRNA back from the common ancestor of eudicots to sometime after the gymnosperm/angiosperm divergence. It should be noted, however, that parallel gains in the magnoliids and eudicots is possible in this case as well. The small size and conserved nature of tRNA genes is such that these competing hypotheses are difficult, if not impossible, to test with phylogenetic analysis.

We know from other angiosperm mitochondrial genomes that sequence transfer from the plastid genome is frequent on an evolutionary timescale [14,55] and that on occasion these transfer events led to the gain of functional tRNAs, based on their widespread conservation across angiosperms [56]. However, the timing of functional transfers has been unclear. Due to its slow rates of gene loss, sequence change and gene-cluster fragmentation, *Liriodendron* may have retained one or more regions of plastid DNA that date back to the original sequence transfers that permanently seeded some of the plastid tRNAs found across angiosperm mitochondrial genomes (Figures 2 and 3). Other interpretations are possible, however. For example, that *Liriodendron* and most eudicots have *trnD(GTC)-cp* (Figures 2 and 3A) could be due to independent parallel gains, once in a magnoliid ancestor and once early in eudicot evolution.

Part of our reasoning that the plastid-derived sequences in Figure 3A, B may be remnants of early functional plastid tRNA transfers is that the tRNA appears to be more strongly conserved than the flanking regions that were simultaneously transferred, suggesting that purifying selection has preserved the tRNA while the surrounding noncoding sequence deteriorated. The fragment in Figure 3A appears to be the oldest, having accumulated 15% pairwise sequence divergence. Given the inferred low rates of sequence evolution in both the mitochondrial and plastid genomes of *Liriodendron*, its transfer may well date to early in angiosperm evolution. We hesitate, however, to estimate the actual timing of the transfer event for several reasons. The current low substitution rates in the magnoliid lineage are possibly lower than rates were earlier in angiosperm evolution, precluding the use of a strict molecular clock. The transferred regions contain plastid sequence with intergenic DNA, as well as synonymous and nonsynonymous sites, which are under different constraints in the plastid relative to the mitochondrial genome, further complicating fragment-wide divergence time estimates. The plastid-derived fragment containing *trnP(TTG)-cp* (Figure 3B) appears to be more recently transferred than the fragment in Figure 3A, given the lower overall divergence from its cognate plastid sequence. In this case, however, more of the fragment consists of protein-coding genes, which would likely decrease the overall rate of pairwise sequence divergence following the transfer event.

Our interpretation of the time since transfer may also be complicated by the possibility that concerted evolution homogenizes homologous plastid and mitochondrial sequences [57]. For example, it is possible that a divergent, plastid-derived sequence fragment containing *trnN(GTT)-cp* (Figure 3C) was already present in the *Liriodendron* mitochondrial genome from an earlier transfer, and the short stretch containing the tRNA was “updated” via gene conversion between it and a reintroduced copy of the same stretch of plastid DNA, restoring the sequence identity between the plastid and mitochondrial copies. This concerted-evolution mechanism was postulated to explain patterns of sequence divergence in a stretch of plastid-derived sequence in the mitochondrial genomes of *Oryza* and *Zea*, where within-species plastid/mitochondrial divergence is less than between species in the mitochondrial region, despite the putatively shared origin of the transferred fragment [57]. If mitochondrial and plastid copies are evolving in concert, the nearly identical plastid-derived fragment in Figure 3C could be much older than suggested by the high sequence similarity.

### Low mitochondrial and plastid substitution rates in magnoliids

The mitochondrial genes in *Liriodendron* evolve at an exceptionally low rate, accumulating just 0.035 nucleotide substitutions per silent site per billion years. As a point of reference, using the same computational approach as employed for the plant mitochondrial rate analysis, we aligned all 13 protein coding genes from the full mitochondrial genomes of a human [58], a Neanderthal [59], a more distantly related Denisova hominin [60], and a chimpanzee outgroup [61]. We calculated an absolute silent substitution rate of 69.5 ssb in humans, using the relevant divergence dates from Krause *et al.*[60]. The human mitochondrial substitution rate is more than 5,000 times faster than *Magnolia* and 2,000 times faster than *Liriodendron*. Stated differently, the average amount of silent site mitochondrial divergence accrued over the course of a single generation (25 years) in humans would take roughly 50,000 years in *Liriodendron* and 130,000 years in *Magnolia*.

Mower *et al.*[10] characterized mitochondrial silent substitution rates across approximately 600 plant species with datasets of one to five genes and also found that *Silene noctiflora* is the fastest [10]. The slowest evolving mitochondrial genome reported by Mower *et al.*[10] was *Cycas* at 0.02 +/− 0.1 ssb, similar to the *Liriodendron* rate reported here, and greater than our estimate for *Magnolia* using an 18-gene concatenated alignment. To our knowledge, the estimated rate of 0.013 ssb in *Magnolia* is the lowest reported genome-wide substitution rate in any organism, but this conclusion is tempered by the associated error in our estimates. For *Magnolia* and *Liriodendron*, the 95% likelihood confidence interval about the ssb estimate due to errors in branch specific synonymous substitution estimation was 0.003 to 0.034 and 0.015 to 0.065, respectively (Additional file 1: Table S3). In addition, our estimates rely heavily on fossil-calibrated divergence times, which add an additional source of error (for example, see [30,31,62,63]). We used two widely accepted fossils within magnoliids [64,65], which together should provide a relatively accurate divergence time estimate for the relevant *Liriodendron*–*Magnolia* split. The 95% highest probability density interval for this split was 94.9 to 102.2 mya, and the median value we used for our estimate was 97.4 mya (see Methods). Therefore, in our study, errors in absolute rate estimation for *Liriodendron* and *Magnolia* are less influenced by divergence time uncertainty than by error in the likelihood estimate of the branch-specific synonymous substitution rates.

We found that mitochondrial and chloroplast substitution rates were roughly correlated in the taxa examined here (Figure 4), an observation deserving of more detailed follow-up study. Although it is too early to extrapolate too much, growth habit (annual vs. perennial, shrub vs. tree) might underlie this pattern [66]. Generation time and rates of synonymous substitution are generally inversely correlated in plants (for review, see [67]). The driving forces behind this relationship are unclear, however, as plants do not have a dedicated germ line, so generation time and number of reproductive cell divisions per year are not as closely linked as they are in animals. Differences between annuals and perennials, in terms of speciation rate and/or metabolism, could underlie the generation time substitution rate relationship [67], and might be expected to similarly influence each of the plant’s three genomes. As nuclear genomic data become available for a broader diversity of plants, it will be interesting to determine whether this correlation extends across all three genetic compartments.

Our data also recovered a greater ratio of plastid to mitochondrial silent substitution rates than was found previously [9,13,51,52]. Our estimate benefited from considerably more sequence data and much broader taxon sampling than previous studies, which might account for the discrepancy. In addition, given the 5,000-fold and 40-fold range in mitochondrial and plastid substitution rates, respectively, that we found, it appears that taxon sampling can have a large effect on average inferred ratios. “High-rate” mitochondrial and plastid lineages do not always have proportionally elevated rates in both organelle genomes [48], leading to extreme plastid–mitochondrial rate relationships (for example, 0.08 in *Silene conica*) (Figure 4). Gene-to-gene variation in mitochondrial [10] and plastid [48,53] silent substitution rates are common as well, underscoring the need to consider many mitochondrial and plastid genes for an accurate determination of relative rates.

### Retention of RNA editing sites lost in many lineages

The overall high level of C-to-U RNA editing in *Liriodendron*, along with its large number of unique edit sites, add further support for a model of relatively high levels of RNA editing in the ancestral angiosperm mitochondrial genome (approximately 700 sites in protein-coding genes), followed by various degrees of subsequent loss in different lineages (Figure 5) [26,27]. RNA editing data from an angiosperm from an “early diverging” lineage, such as *Amborella* or *Nymphaea*, would help polarize the degree of editing loss in *Liriodendron*, which looks to be exceptionally low based on these data. There is no clear adaptive explanation for the emergence and maintenance of RNA editing in plants [25,68,69], but it may have emerged through neutral processes, only to become essential following substitutions at functionally important cytosines that required post-transcriptional editing to produce the conserved amino acid [70] – a hypothesis falling under the category of ‘constructive neutral evolution’ [71,72]. Consistent with this model, most edit sites change the translated amino acid sequence [21,73], a pattern underscored in *Liriodendron*, in which 82% of the edits were at nonsynonymous sites. While the emergence of RNA editing may be due to neutral processes, comparative work has found support for selection favoring loss of editing over time [26,27], and it is likely that such selection would be stronger at nonsynonymous sites, where unreliable editing would be most deleterious. Consistent with this hypothesis, we found the ratio of loss to gain was 14:1 at nonsynonymous sites compared to 2:1 at silent sites across angiosperms (Figure 5).

### Conservation of ancient gene clusters

Although overall gene order is highly variable among angiosperm mitochondrial genomes [13], even between closely related taxa [15], the results here underscore countervailing constraints on short clusters of gene linkage operating across angiosperm evolution. While some of the conserved clusters (for example, *rrnS–rrn5* and *rpl2–rps19–rps3–rpl16*) date back to the original bacterial ancestor of mitochondria [19], others are unique to angiosperms, such as the *atp8–cox3–sdh4* and *rps13–nad1.x2.x3* clusters. The five clusters shared by *Liriodendron* and *Cycas* most likely were present early in seed plant evolution, and we can look outside of seed plants to infer which of these were also present early in vascular plant evolution as well. A comparative gene order analysis showed *Huperzia* to have experienced fewer rearrangements relative to bryophytes than any other vascular plant mitochondrial genome [74], making it a meaningful comparison for vascular plant gene order conservation. Of the five clusters shared by *Cycas* and *Liriodendron*, three are shared with *Huperzia* and two are not. All of the gene clusters found in *Liriodendron* to the exclusion of *Cycas* are also lacking in *Huperzia*, suggesting such clusters are indeed angiosperm-specific.

Transcription is likely an important constraint, whereby adjacent genes share a single promoter and are co-transcribed, as was shown for three conserved gene clusters in *Nicotiana*[16]. This could explain why all of the clusters conserved across angiosperms involve genes encoded on the same strand. Interestingly, three of the clusters inferred to be present in the ancestral angiosperm involve internal fragments of *trans*-spliced genes (Figure 6), which may, upon further examination, provide clues as to the regulation and reconstruction of full-length transcripts from *trans*-spliced genes.

The *Liriodendron* mitochondrial genome appears to have been subject to both low silent-substitution rates and infrequent gene-cluster fragmentation relative to sequenced eudicot and monocot mitochondrial genomes (Figures 4 and 6). However, levels of silent substitution and gene cluster fragmentation do not necessarily covary across all angiosperms in our study. For example, one of the taxa with a relatively high silent substitution rate (>30 × faster than *Liriodendron*), *Cucurbita*, has 11 conserved gene clusters compared to 12 in *Liriodendron,* whereas *Zea*, with a relatively slower rate (10 × faster than *Liriodendron*), has only five. In angiosperm plastid genomes, there is support for a positive relationship between rates of structural and sequence evolution [75], but this relationship is not universal [48,53]. In *Silene*, for example, although rates of plastid gene order rearrangement are higher in species with higher substitution rates, many of these substitutions occur at nonsynonymous sites and so are not easily explained by a simple, mutationally-driven model [48].

## Conclusions

Although nearly 40 flowering plant mitochondrial genomes have been sequenced to date, the addition of *Liriodendron*, because of its phylogenetic position and extraordinary level of sequence and gene-cluster conservation, greatly refines our view of the ancestral angiosperm mitochondrial genome. This, in turn, provides valuable insights into patterns of mitochondrial genome evolution across angiosperms. The ancestral angiosperm mitochondrial genome almost certainly contained 41 protein-coding genes, most likely possessed 14 native mitochondrial tRNAs, including the *trnV(TAC)* described here, and perhaps harbored as many as seven functional plastid-derived tRNA genes. Several tRNA-containing plastid-derived sequences in the *Liriodendron* mitochondrial genome may trace all the way back to the original functional transfers of these plastid tRNAs, which became permanent fixtures in angiosperm mitochondrial genomes. *Liriodendron* contains 12 gene clusters that appear to have been maintained from the ancestral angiosperm genome. *Liriodendron* encodes 75% of the superset of angiosperm RNA editing sites, consistent with high levels of editing in the ancestral angiosperm, followed by a progressive pattern of loss over time.

Genomic data from additional “early diverging” angiosperms, such as *Nymphaea* and *Amborella*, will provide more detailed insights into mitochondrial genome evolution in early angiosperms. We now have one sequenced magnoliid mitochondrial genome, but there are still three major unsampled lineages (Laurales, Piperales and Canellales), so we cannot yet know how representative *Liriodendron* is of magnoliids as a whole. However, it appears from the limited comparative data presented here that the magnoliids might exhibit uniquely low levels of genomic change.

## Methods

### Mitochondrial DNA isolation and sequencing

A modified Sandbrink high-salt isolation [76] with DNase I treatment was used to prepare the purified mitochondrial DNA for sequencing. A total of 200 grams of fresh young leaves were harvested from a single *Liriodendron tulipifera* tree (AR501) from the campus of Indiana University in Bloomington, Indiana. All extraction steps were carried out in a cold room at 4°C unless otherwise noted. Washed leaves were homogenized in one liter of high-salt homogenization buffer (1.25 M NaCl, 50 mM Tris–HCl, 5 mM EDTA, 5% w/v PVP-40, 1% w/v BSA, 15 mM β-mercaptoethanol) in a Waring blender (Waring Laboratory Science, Torrington, CT, USA), and the homogenate was filtered through four layers of cheesecloth, then one layer of Miracloth (EMD Millipore, Billerica, Massachusetts, USA).

The filtrate was centrifuged twice at 2,000 × *g* for 10 minutes. The supernatant was recovered and centrifuged for 15 minutes at 12,000 × *g* to collect the mitochondria. The mitochondrial pellet was gently resuspended in 5 mL of DNase I buffer (0.35 M sorbitol, 50 mM Tris–HCl, 15 mM MgCl_2_) followed by the addition of 30 mg dry DNase I. The DNase I digestion was carried out on ice for one hour, with occasional swirling, and stopped by the addition of three volumes of wash buffer (0.35 M sorbitol, 50 mM Tris–HCl, 25 mM EDTA). The DNase I-treated mitochondria were centrifuged for 20 minutes at 12,000 × *g* and resuspendend a total of three times, with a final resuspension in 1 mL wash buffer and 1/5 volume lysis buffer (5% w/v sodium sarcosinate, 50 mM Tris–HCl, 25 mM EDTA). Lysis was carried out at 37°C for 15 minutes. DNA from the lysate was separated in a 24:1 mixture of chloroform and isoamyl alcohol, and precipitated with the addition of 0.1 volumes of sodium acetate and 2 volumes of 95% ethanol overnight at −20°C. The pellet was resuspended in 100 μL TE. Purity was assessed with standard DNA gel blots [77] with mitochondrial (*matR*), plastid (*rbcL*) and nuclear (18S rDNA) PCR probes, amplified from a total DNA isolate of *L. tulipifera*.

The genome was sequenced using standard Sanger sequencing (12 × depth) and Roche/454 (Roche Applied Science, Basel, Switzerland) GS FLX pyrosequencing (60 × depth). The Sanger sequencing was done at the Joint Genomes Institute (JGI) in Walnut Creek, California, and pyrosequencing was done at the Indiana University Center for Genomics and Bioinformatics.

### Genome assembly and annotation

The pyrosequencing reads initially were assembled with Roche’s Newbler assembler (ver. 1.0.53.17). Sanger reads and pyrosequencing contigs initially were assembled with CAP3 [78], then manual assembly and in-house scripts were used to piece the contigs together with the Sanger read-pair information. The final assembly was verified by aligning all data to it and evaluating high quality mismatches inside and outside the aligned regions. Read-pair depth in repeats was evaluated using lastz and samtools as implemented by Galaxy [79]. Features were annotated manually based on the output of NCBI-BLASTN and -BLASTX searches to custom databases.

Coverage by annotated features (protein coding genes, *cis*-spliced introns, rDNAs, tRNAs, plastid-like sequences and repeats >500 bp in length) was tallied, and these regions were then masked from subsequent analyses. To find intergenic regions shared with other plant mitochondrial genomes, the masked sequence file was used as a query against a database of the currently available streptophyte mitochondrial genomes using BLASTN 2.2.24+ with default parameters and an e-value cutoff of 10. All regions found with mitochondrial hits using these conditions were masked. To identify small repeats, the remaining masked sequence was used as subject and query using BLASTN 2.2.24+, with word_size = 7 and no ‘dust’ filter. Finally, the remaining sequence was submitted to repeat-masker (http://www.repeatmasker.org), with default parameters to identify transposon-like sequences. The nucleotides remaining after this iterative masking process were counted in the ‘unknown intergenic’ category. Due to the overlapping nature of the categories (for example, a large repeat can contain a gene), the sum across all categories exceeds the genome size by 22 kb.

### cDNA sequencing

RNA was isolated from one gram of fresh *L. tulipifera* (AR501) leaf tissue using a lithium acetate extraction protocol [80]. Following LiAc precipitation, the RNA pellet was dissolved in 50 μL of water and DNase treated with TURBO DNase (Life Technologies, Carlsbad, CA, USA). DNase-treated total RNA samples were reverse transcribed (RT) using the Omniscript RT kit (Qiagen, Hilden, Germany). A set of random nonamers were used with approximately 2 μg of total RNA in a 20 μL volume RT reaction. A control reaction without reverse transcriptase was conducted to ensure there was no DNA contamination in the RNA sample and RT reagents.

PCR was conducted for 38 protein-coding genes, for a total of 30,327 bp. PCRs were amplified in a 50 μL reaction volume using NEB reagents and gene-specific primer combinations at a concentration of 0.4 μM (primers and annealing temperatures available upon request). Amplified products were purified using the QIAquick PCR purification kit (Qiagen) and directly sequenced on an ABI 3730 (Applied Biosciences, Inc., Foster City, CA, USA). RNA editing was assessed by aligning the cDNA sequences to the genomic sequence.

### Magnoliid PCR and DNA sequencing

Total DNA was extracted from 1 g of leaf tissue harvested from a *Magnolia stellata* tree on the Indiana University campus using a standard cetyltrimethyl ammonium bromide (CTAB) protocol [81]. PCR and ABI 3730 Sanger sequencing were conducted for 20 mitochondrial genes using standard protocols. An additional gene sequence for *Calycanthus floridus* (YQ 94155) was generated using primers and conditions described previously [82].

### Substitution rates

In-frame DNA alignments of the gene sequences were made using the translated amino-acid sequence using BioEdit 7.0.9.0 [83] and ClustalW [84] and adjusted by eye. Low quality regions were masked from subsequent analyses. cDNA sequences were determined empirically for *Liriodendron*, whereas edit sites from the remaining 10 taxa (Figure 4) were parsed from GenBank or REDIdb. Codons with an edited nucleotide in any of the 11 taxa were masked, as were stop codons. Apparent pseudogenes (with frameshifts or premature stop codons) were not included in these analyses.

Using a constrained topology consistent with the current consensus understanding of angiosperm phylogeny [33,34] and a concatenated mitochondrial 18-gene alignment (*atp1, atp6, ccmB, ccmC, ccmFN, cob, cox1, cox3, matR, nad1, nad2, nad3, nad4, nad5, nad7, nad9, rps3, rps4*; 16,305 bp in total), maximum likelihood estimates of branch-specific silent substitution rates were calculated with HyPhy ver. 2.0 [85] under the MG94W9 model [86]. The same analysis was conducted with a concatenated alignment of the *atpB*, *matK* and *rbcL* genes (3,936 bp in total) with the same taxa to infer branch-specific silent substitution rates in the plastid genome. Confidence intervals of 95% about the branch-specific rates were estimated using the ‘Likelihood Profile’ method in HyPhy, which is based on the quadratic approximation to the likelihood surface.

To scale the substitutions per site to absolute substitution rates, a chronogram was estimated using the same 13 taxa and a concatenated three-plastid-gene alignment (3,936 bp) with BEAST ver. 1.61 [87] and the following parameters: the Yule model of speciation, an uncorrelated relaxed clock, and the GTR substitution model with four gamma rate parameters. The topology was constrained as above, and four age constraints were used at the following nodes: the common ancestor of eudicots (125 million year offset, lognormal prior with a mean of 1.5 and standard deviation of 0.5, based on the widely used calibration [30,31], derived from the earliest observation of fossil tricolpate pollen in the Barremian–Aptian [88]); the common ancestor of grasses (normal prior with a mean of 51.6 million years and a standard deviation of 3, an approximation of the estimate made by Vicentini *et al.*[89]); the common ancestor of *Liriodendron* and *Magnolia* (93.5 million year offset, lognormal prior with a mean of 1.5 and standard deviation of 0.5, from a fossil constraint in Frumin and Friis [64]); and the common ancestor of the Magnoliaceae and Calycanthus (108.8 million year offset, lognormal prior with a mean of 1.5 and standard deviation of 0.5, from a fossil constraint in Friis *et al.*[65]). The simulation was run for 50 million rounds, with output logged every 50,000 generations. The first 100 trees were discarded (10% burn-in), and the remainder was used to calculate the median ages and 95% highest probability density intervals of the nodes in the tree using TreeAnnotator v1.61 [87]. The absolute substitution rates were calculated by dividing the HyPhy branch substitution rates by the BEAST median estimate of branch age for each branch in the tree. In the case of *Silene vulgaris*, *S. noctiflora* and *S. conica*, rate estimates were taken from previously published reports [2,48] and incorporated into our analysis using an estimate of 6 mya at the base of the *Silene* polytomy. These three taxa were not included in our mitochondrial or plastid concatenated gene alignments for rate or chronogram estimation.

To assess silent substitution rates within the genus *Magnolia*, we calculated pairwise silent substitutions per site in alignments across six genes (*atp1*, *nad3*, *nad5*, *rps4, rps12, rps13*; 5,718 bp in total) for which multiple high-quality *Magnolia* sequences were available: *M. figo* [*atp1*: AY299802], *M. grandiflora* [*atp1*: AF209100, *nad3*: Z49773, *rps12*: Z49773], *M. tripetala* [*atp1*: AF197691, *nad5*: DQ406916] *Magnolia x soulangeana* [*nad3*: Z49797, *rps4*: AF375592, *rps12*: Z49797, *rps13*: Z49798], and *M. stellata* [*nad5*, *rps4*, *rps13*, this study]. To assess the same within the genus *Liriodendron*, we calculated pairwise silent substitutions per site for four genes (*atp1*, *matR*, *nad5*, *rps3*; 7,098 bp) for which sequence was also available for its congener, *L. chinense* (AF197690, AF197774, DQ406926, GU351719) relative to *L. tulipifera* (this study).

To examine the plastid derived sequence flanking several tRNAs of interest, we aligned the plastid and mitochondrial sequences and a created sliding window plots with Synplot (http://hscl.cimr.cam.ac.uk/syn_plot.html) with a 25 bp window and a 10 bp sliding increment. With this tool, indels count as mismatches in the calculation of the percent identity between the two sequences.

### RNA editing

Alignments of edited sequences were made as described above. Each column containing at least one edited residue in the alignment was transformed into a column in a presence/absence matrix, with ‘0’ indicating no editing, ‘1’ indicating editing, and ‘?’ denoting missing data. Regions of the alignments for which we had no *Liriodendron* cDNA sequence were omitted from the analysis. The number of edit sites included for each species is shown in Additional file 1: Table S2. The most parsimonious scenario of gains and losses along the branches of the tree was calculated using the Count software [90], under Dollo parsimony, which does not allow parallel gains at the same site. Edit sites were subsequently partitioned by whether they occur at a nonsynonymous or synonymous site. Edit sites in codons with multiple edits or where *Liriodendron* encoded a G or A were omitted in the partitioned data. As a result, the partitioned sites sum to fewer than the total number of sites in the study.

### Gene cluster analysis

A set of in-house Perl scripts were used to parse the presence of clusters of genes in available mitochondrial genomes for *Cycas* and angiosperms. A cluster is defined as two or more adjacent genes separated by fewer than 5 kb of intergenic sequence and with no intervening genes. The relative orientation of the adjacent genes was also noted (that is, the same or opposite strand). These clusters were compared across species and recorded if they were present, in the same relative orientation, in at least *Cycas* or *Liriodendron* plus a monocot or eudicot. If a cluster was not present in *Cycas* or *Liriodendron*, it was shown only if shared between a monocot and a eudicot, or between a grass and a non-grass monocot, or between an asterid and a rosid.

### tRNA evolution

In order to reconstruct the history of plastid-derived tRNA gain and loss in angiosperms, presence or absence (as determined by BLAST and manual inspection) in the mitochondrial genomes in this study was mapped to the current consensus understanding of angiosperm phylogeny [33,34]. Gains and losses were inferred by parsimony. In the case of equally parsimonious scenarios, only one is shown, but no assumption was made about the relative probabilities of gain or loss. To be included in the analysis, a plastid-derived tRNA had to be present in *Cycas* or *Liriodendron* plus a monocot or eudicot. If the tRNA was not present in *Cycas* or *Liriodendron*, it was shown only if shared between a monocot and a eudicot, or between a grass and a non-grass monocot, or between an asterid and a rosid.

## Abbreviations

DR: Direct repeat; IR: Inverted repeat; JGI: Joint Genomes Institute; mya: Million years ago; ssb: Silent substitutions per site per billion years

## Competing interests

The authors declare that they have no competing interests.

## Authors’ contributions

AOR and GJY conducted the molecular biology experiments. DWR assembled the genome. DWR, AOR and AJA annotated the genome. AOR, AJA and JDP analyzed the data and wrote the manuscript. All authors read and approved the manuscript.

## Supplementary Material

Additional file 1: Table S1 Content of the 553,721 bp mitochondrial genome of *Liriodendron tulipifera*. **Table S2.** Empirically determined RNA editing sites in each of the 10 species in the study broken down by gene. ‘ND’ indicates no data available, and the total number of unique edit sites across each gene given at bottom. See Methods for data sources. **Table S3.** Estimated silent substitutions per site per billion years (ssb) and associated 95% confidence interval (CI) lower and upper bounds for the taxa in Figure 4 (other than the *Silene* species) for both the concatenated mitochondrial (mt) and plastid (cp) gene alignments. **Figure S1.** Current consensus cladogram of relationships among the eight major lineages of angiosperms [34]. Numbers above branches are estimates of the number of extant species in each group as reported by APG III [33]. **Figure S2.** Genome content across seed plants. The cladogram at left is based on current phylogenetic consensus [33,34] and the scale at right is in kb. Genome size, in kb, for each sample is given in the middle column. Click here for file

## Data depositions

All sequences have been deposited to GenBank [accession numbers: KC821969 and KC879625-KC879661].
