# Supplementary material for: The “fossilized” mitochondrial genome of Liriodendron tulipifera: ancestral gene content and order, ancestral editing sites, and extraordinarily low mutation rate
Source: BMC Biol. 2013 Apr 15;11:29. doi: 10.1186/1741-7007-11-29 (PMC3646698; doi:10.1186/1741-7007-11-29)
Supplement: Additional file 1: Table S1 — Content of the 553,721 bp mitochondrial genome of Liriodendron tulipifera. Table S2. Empirically determined RNA editing sites in each of the 10 species in the study broken down by gene. ‘ND’ indicates no data available, and the total number of unique edit sites across each gene given at bottom. See Methods for data sources. Table S3. Estimated silent substitutions per site per billion years (ssb) and associated 95% confidence interval (CI) lower and upper bounds for the taxa in Figure 4 (other than the Silene species) for both the concatenated mitochondrial (mt) and plastid (cp) gene alignments. Figure S1. Current consensus cladogram of relationships among the eight major lineages of angiosperms [34]. Numbers above branches are estimates of the number of extant species in each group as reported by APG III [33]. Figure S2. Genome content across seed plants. The cladogram at left is based on current phylogenetic consensus [33,34] and the scale at right is in kb. Genome size, in kb, for each sample is given in the middle column. [file 1741-7007-11-29-S1.pdf]

Table S1. Content of the 553,721 bp mitochondrial genome of *Liriodendron tulipifera*.

Category	Feature	#	bp (%)†
**Genes**			
	protein coding	41	34,499 (6.2)
	pseudogenes	2	670 (0.1)
	rRNA	3	5,883 (1.1)
	mt-derived tRNA	13	996 (0.2)
	cp-derived tRNA	7	443 (0.1)
**Introns**			
	cis-spliced	20	36,353 (6.6)
	trans-spliced	5	n.d.
**Intergenic**			
	mt-derived*	n.d.	128,887 (23.3)
	cp-derived	25	26,887 (4.9)
	repeats > 500 bp	10	51,615 (9.4)
	repeats < 500 bp	n.d.	29,713 (5.4)
	transposon-like	n.d.	5,940 (1.1)
	unknown	n.d.	254,098 (45.9)

* includes *trans*-spliced introns

† sum across categories exceeds 100.0% due to overlapping features such as genes in repetitive regions; see Methods.

	atp1	*atp4*	*atp6*	*atp8*	*atp9*	*ccmB*	*ccmC*	*ccmFc*	*ccmFN*	*cob*
Arabidopsis	5	8	1	ND	4	39	28	16	33	7
Brassica	4	8	1	3	4	39	25	ND	24	8
Oenothera	3	ND	22	6	4	46	28	18	44	13
Citrullus	7	13	22	3	7	34	16	11	ND	13
Cucurbita	3	12	20	3	6	33	17	13	ND	13
Beta	3	12	11	2	5	30	28	13	22	13
Silene	3	11	11	2	4	27	23	12	ND	9
Nicotiana	6	10	15	5	10	46	30	18	24	12
Oryza	4	9	15	3	8	35	34	27	ND	19
Vitis	8	5	17	6	4	6	15	6	ND	25
Liriodendron	14	16	29	6	15	49	41	24	34	30
**Edit Sites**	**18**	**21**	**35**	**15**	**17**	**71**	**53**	**68**	**51**	**45**

	cox1	*cox2*	*cox3*	*matR*	*mttB*	*nad1*	*nad2*	*nad3*	*nad4*	*nad4L*
Arabidopsis	ND	9	8	9	24	21	31	10	32	9
Brassica	1	8	7	8	27	20	24	8	35	9
Oenothera	ND	14	ND	8	20	24	35	13	ND	ND
Citrullus	18	9	9	4	21	20	26	12	43	13
Cucurbita	18	10	6	4	27	19	25	9	33	13
Beta	ND	8	4	9	19	18	23	10	19	9
Silene	ND	2	1	8	15	17	20	7	16	8
Nicotiana	13	12	8	11	43	22	28	15	37	13
Oryza	4	15	ND	ND	33	19	29	13	20	10
Vitis	22	16	10	11	ND	12	18	3	21	7
Liriodendron	37	14	19	19	58	33	49	21	58	13
**Edit Sites**	**42**	**23**	**23**	**23**	**72**	**45**	**64**	**32**	**78**	**17**

	nad5	*nad6*	*nad7*	*nad9*	*rpl2*	*rpl5*	*rps10*	*rps12*	*rps13*	*rps14*
Arabidopsis	27	10	22	7	1	10	ND	8	ND	ND
Brassica	29	11	23	7	1	9	ND	7	ND	ND
Oenothera	26	ND	ND	ND	4	9	ND	ND	3	2
Citrullus	29	ND	26	7	1	9	5	6	3	ND
Cucurbita	21	ND	25	7	1	8	5	6	3	ND
Beta	17	11	16	5	ND	5	ND	6	2	ND
Silene	18	10	15	5	ND	6	ND	ND	ND	ND
Nicotiana	24	1	23	7	ND	7	ND	4	3	ND
Oryza	11	16	28	11	ND	1	ND	ND	8	ND
Vitis	21	9	19	7	1	13	7	8	6	3
Liriodendron	49	25	39	14	3	12	6	9	7	2
**Edit Sites**	**59**	**34**	**45**	**16**	**7**	**16**	**7**	**14**	**11**	**3**

	rps19	*rps3*	*rps7*	*sdh3*
Arabidopsis	ND	13	ND	ND
Brassica	ND	8	1	ND
Oenothera	7	13	ND	ND
Citrullus	3	7	2	3
Cucurbita	4	10	3	5
Beta	ND	6	2	ND
Silene	ND	4	ND	ND
Nicotiana	5	10	ND	1
Oryza	6	10	2	ND
Vitis	8	9	3	1
Liriodendron	5	17	4	10
**Edit Sites**	**13**	**31**	**5**	**12**

Table S2. Empirically determined RNA editing sites in each of the 10 species in the study broken down by gene. 'ND' indicates no data available, and the total number of unique edit sites across each gene given at bottom. See Methods for data sources.

	mt ssb	95% CI lower	95% CI upper	cp ssb	95% CI lower	95% CI upper
Arabidopsis	0.980	0.825	1.155	4.739	3.966	5.616
Carica	0.257	0.180	0.352	1.552	1.122	2.072
Vigna	0.775	0.642	0.924	6.931	5.929	8.057
Cucurbita	1.219	1.050	1.407	3.215	2.573	3.950
Vitis	0.178	0.123	0.249	0.918	0.695	1.182
Beta	0.964	0.837	1.102	3.406	2.872	4.011
Nicotiana	0.489	0.402	0.589	2.497	2.048	3.011
Zea	0.335	0.241	0.451	2.304	1.681	3.036
Oryza	0.290	0.202	0.399	2.283	1.660	3.016
Liriodendron	0.035	0.015	0.065	0.175	0.085	0.310
Magnolia	0.013	0.003	0.034	0.198	0.103	0.337
Calycanthus	0.120	0.084	0.167	1.091	0.841	1.384

Table S3. Estimated silent substitutions per site per billion years (ssb) and associated 95% confidence interval (CI) lower and upper bounds for the taxa in Figure 4 (other than the *Silene* species) for both the concatenated mitochondrial (mt) and plastid (cp) gene alignments.

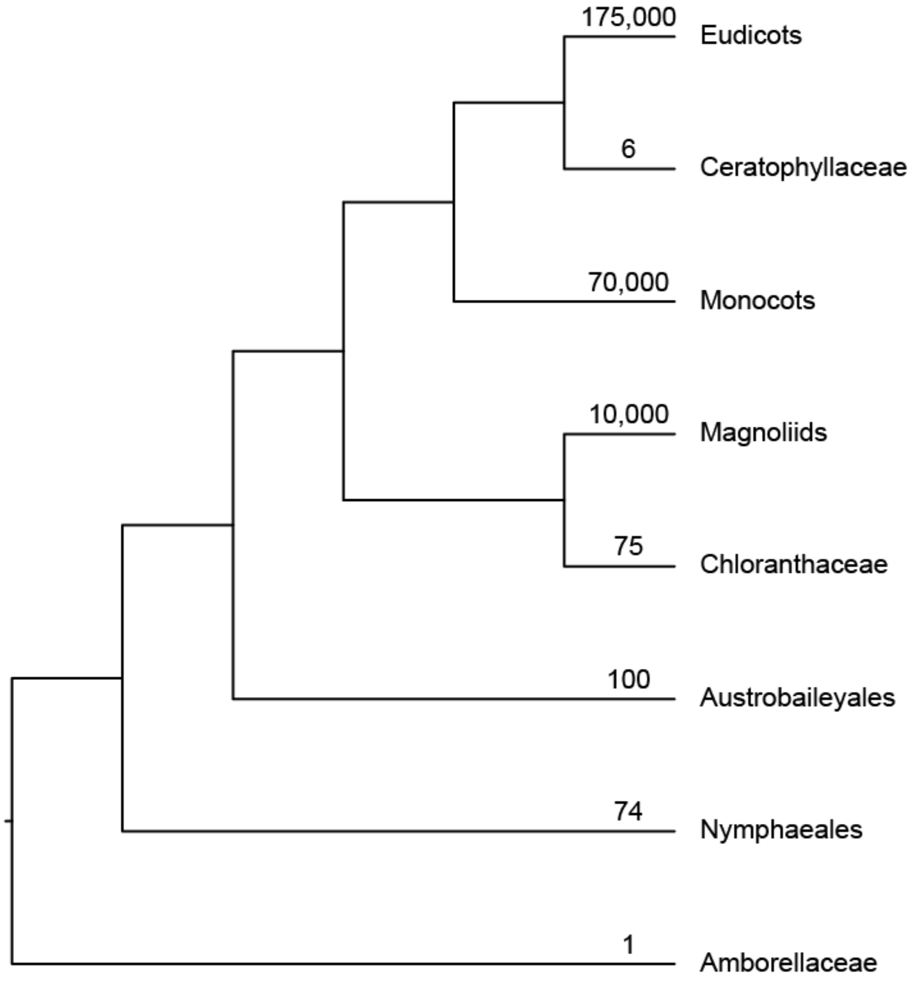

Figure S1. Current consensus cladogram of relationships among the eight major lineages of angiosperms [1] . Numbers above branches are estimates of the number of extant species in each group as reported by APG III [2].

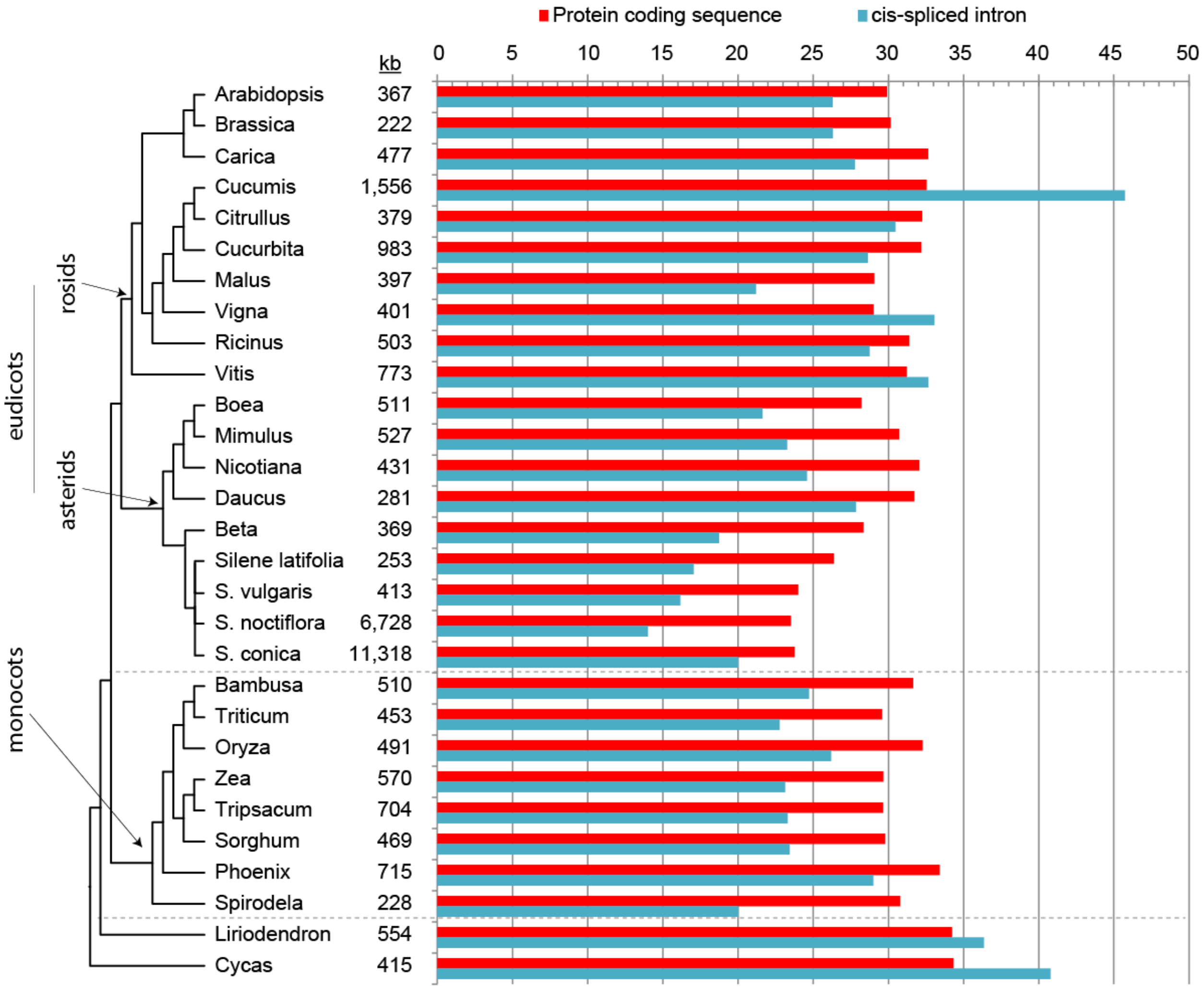

Figure S2. Genome content across seed plants. The cladogram at left is based on current phylogenetic consensus [1, 2] and the scale at right is in kb. Genome size, in kb, for each sample is given in the middle column.

1. Soltis DE, Smith SA, Cellinese N, Wurdack KJ, Tank DC, Brockington SF, Refulio-Rodriguez NF, Walker JB, Moore MJ, Carlsward BS *et al*: **Angiosperm phylogeny: 17 genes, 640 taxa**. *Am J Bot* 2011, **98**(4):704-730.
2. APG3: **An update of the Angiosperm Phylogeny Group classification for the orders and families of flowering plants: APG III**. *Bot J Linn Soc* 2009, **161**(2):105-121.
